# Differential Protein-Coding Gene Expression Profile in Patients with Prostate Cancer

**DOI:** 10.3390/biomedicines12112509

**Published:** 2024-11-01

**Authors:** Lorena Albarracín-Navas, Maylin Almonte-Becerril, Enmanuel Guerrero, Josue Rivadeneira, Marcelino Telechea-Fernández, Elizabeth Guzmán, Fanny Calderón, María José Hernández-Leal, Tamara Otzen, Carlos Manterola, Galo Duque, Ángela L. Riffo-Campos

**Affiliations:** 1 Universidad de La Frontera, Ph.D. Program in Medical Sciences, Temuco 4811230, Chile; l.albarracin01@ufromail.cl (L.A.-N.); enmanuel.guerrero@ucuenca.edu.ec (E.G.); j.rivadeneira01@ufromail.cl (J.R.); mhernandezl.1@unav.es (M.J.H.-L.); tamara.otzen@ufrontera.cl (T.O.); carlos.manterola@ufrontera.cl (C.M.); 2 Universidad de La Frontera, Millennium Nucleus on Sociomedicine (SocioMed), Temuco 4811230, Chile; 3 Comprehensive Medical Services (SERMEDIC), Cuenca 010111, Ecuador; 4 Executive Direction of Research and Advanced Studies, Universidad de la Salud, Mexico City 01210, Mexico; maylin.almonteb@unisa.cdmx.gob.mx; 5 Faculty of Medicine, Universidad de Cuenca, Cuenca 010201, Ecuador; 6 SOLCA Cancer Institute, Cuenca 010111, Ecuador; 7 Zero Biomedical Research, Quito 170103, Ecuador; 8 Faculty of Chemistry, Complutense University of Madrid, 28040 Madrid, Spain; marcetel@ucm.es; 9 University of Navarra, School of Nursing, Department of Community, Maternity and Pediatric Nursing, 31006 Pamplona, Spain; 10 IdiSNA, Navarra Institute for Health Research, 31008 Pamplona, Spain; 11 Center for Cancer Prevention and Control (CECAN), Santiago 8380453, Chile; 12 Universidad de La Frontera, Center for Morphological and Surgical Studies (CEMyQ), Temuco 4811230, Chile; 13 Faculty of Medicine, Universidad del Azuay, Cuenca 010107, Ecuador; galoduque@uazuay.edu.ec

**Keywords:** prostate cancer, molecular targets, gene expression, mRNAs

## Abstract

Background: Prostate cancer is the second most common neoplasm in men, with projections estimating over one million new cases by 2045. Differentially expressed genes can significantly enhance the diagnosis, treatment, monitoring, and prognosis of this disease. Purpose: to systematically review and analyze validated differentially expressed mRNAs in prostate cancer patients to propose a robust molecular profile for clinical diagnostics. Methods: A systematic review was conducted following PRISMA guidelines, searching literature databases for mRNAs with validated differential expression in adult prostate cancer patients. Identified mRNAs were analyzed using STRING, Cytoscape, and DrugBank to explore protein–protein interactions and potential drug targets. Results: A total of 5003 participants from Europe, Asia, America, and Oceania were included, and 144 mRNAs (*p* < 0.05) were reported across 75 primary articles, predominantly validated using RT-qPCR with tissue samples. Among these, at least 36 mRNAs were identified as targets for cancer-related drugs. Enrichment analysis revealed the top pathways were associated with cancer, including specific prostate cancer terms. Key nodes emerged as hubs in the protein–protein interaction network. Conclusion: Based on our comprehensive in silico analysis of validated differentially expressed mRNAs, we propose a molecular profile of twenty-five mRNAs with significant potential for clinical diagnosis of prostate cancer. These findings offer a valuable foundation for developing precision oncology strategies to improve patient outcomes.

## 1. Introduction

Prostate cancer (PCa) is the second most common malignancy affecting men, with a worldwide male incidence of 14.2% and a mortality of 7.3% [1]. Risk factors mainly include advanced age, family history, and genetic predisposition [2]. Diagnosis is based on clinical evaluation, plasma levels of prostate-specific antigen (PSA) and its free fraction, imaging studies, and prostate tissue biopsy, which remains the standard of care for diagnosis [3]. However, PCa screening has become increasingly precise, with the implementation of newer approaches such as biomarkers [2,3]. A biomarker is an indicator of a biological state, which when it is composed of DNA, RNA, proteins, or their derivatives and/or variations, is called a molecular biomarker. Numerous molecular biomarkers have been proposed for the clinical management of PCa [4]. Most of the commercial molecular biomarkers are genetic, i.e., DNA mutations, and focus on the hereditary origin of PCa, which corresponds to 10% of cases [5].

On the other hand, the traditional PCa treatment, which includes surgical resection, radiotherapy, and hormone therapy, has also improved with the incorporation of molecular targeted therapy, especially in metastatic PCa [6]. The molecular target, unlike the biomarker, has a function that can modify the disease’s course. For instance, transcription factors (like the androgen receptor) are therapeutic targets for prostate cancer, since medications such as enzalutamide and bicalutamide are chemical inhibitors targeting the AR ligand binding domains [7,8].

Therefore, the identification of new molecular profiles represents an opportunity to contribute to the detection and treatment of PCa. In this sense, RNAs with differential expression are emerging as an alternative for clinical diagnosis and target therapy. Tumors can be classified into molecular subtypes based on the mRNA profile, and these molecular subtypes can predict treatment response [9]. The evidence in this regard comes from studies with cell lines [10] or other study models [11], but it is unknown how many of these findings are supported by high-quality evidence. Thus, to understand the gap between basic research on mRNAs with differential expression in patients with PCa and its potential clinical use, it is necessary to compile the evidence and verify those findings directly related to patients. Therefore, we systematically reviewed the primary articles to find the mRNAs with differential expression validated in adults with prostate cancer, and subsequently conducted an in silico analysis to identify the molecular pathways, hub nodes, and drug-related data, which allow us to propose a protein-coding gene expression profile in patients with prostate cancer.

## 2. Materials and Methods

### 2.1. Study Design: Systematic Review

This study was written following the recommendations proposed by the Preferred Reporting Items for Systematic Reviews and Meta-Analysis (PRISMA 2020) statement [12]: registration number PROSPERO CRD42022303584.

#### 2.1.1. Eligibility Criteria

The selected primary articles, published up to 24 September 2023, correspond to studies that include samples of cases and controls in the adult population, including men over 18 years of age with prostate cancer, that reported RNAs with differential expression associated with the diagnosis of this disease. In the case of articles that include transcriptomics data, only those that have their validation cohort were included. In vivo, in vitro, and in silico studies without a validation cohort were excluded. Similarly, gray literature, clinical trial protocols, systematic and other reviews, consensus documents, short communications, opinion pieces, letters to the editor, posters, conference proceedings, series, and case reports were excluded. Articles on patients with metastases, differentially expressed genes related to progression, method studies, and bioinformatics tools were also excluded. When the full text of the study was not available online, attempts were made to contact the corresponding author and libraries, and when this was not possible, the study was excluded.

#### 2.1.2. Information Sources and Search Strategy

The systematic search was carried out in bibliographic databases Medline (by PubMed), Web of Science, Scopus, and Embase. Additionally, a broader search was performed, and the term diagnosis was deleted; this, along with a manual search in the references of the selected articles, allowed us to include related articles not found in the initial search. The search strategy in the bibliographic databases included free and controlled terms (MeSH), descriptors (DeSH), and Boolean operators AND, OR, and NOT (see Supplementary File, Sheet S1: Search Strategy).

#### 2.1.3. Selection of Articles

The authors (L.A.-N. and A.L.R.-C.) carried out an absolute, specific, independent and masked search in the bibliographic databases; discrepancies that arose were resolved by consensus. Duplicate articles were identified and removed (see Supplementary File, Sheet S2). Subsequently, the researchers (L.A.-N. and M.T.-F.) performed the selection by screening the title and abstract of the articles according to the inclusion criteria, discarding those that did not meet at least one of these. When a discrepancy arose between the two reviewers, a third party (A.L.R.-C.) participated and determined the final status of the article. Subsequently, 6 reviewers (L.A.-N., E.G. (Elizabeth Guzmán), G.D., M.A.-B., F.C. and E.G. (Enmanuel Guerrero)) read the full text, and any disagreements were resolved by a seventh reviewer (A.L.R.-C.), who issued the final decision.

#### 2.1.4. Data Collection Process

A digital form was created using Microsoft Excel (version 15.24; 2016 Microsoft Corporation), and the relevant information from the articles with the variables under study was incorporated (see Supplementary File, Sheet S6).

The following variables were studied: The design of the article, the country where the study was carried out, statistical methods, RNA analysis technique, biological sample, and surgical treatment performed on the participants. A summary was prepared with the information from each article, and among the extracted data were the digital object identifier (DOI), author, year of publication, *p*-value, Fold Change, population characteristics (number of cases and controls as well as total number of patients), age, and the gene symbol. The information was retrieved independently by three reviewers (L.A.-N., M.A.-B., and A.L.R.-C.), and differences were agreed upon.

#### 2.1.5. Risk of Bias in Individual Studies

Two independent researchers (J.R. and M.J.H-L.) evaluated the quality of the primary studies, applying the Standard Quality Assessment Criteria for Evaluating Primary Research Papers instrument, from a variety of fields (2004), which has 14 items written in the form of questions; however, 4 do not apply to translational studies. The score ranged from 0, which indicated the lowest level of quality, to 1, which was the highest level [13] (see Supplementary File, Sheet S7).

### 2.2. Molecular Pathway

#### 2.2.1. Drug Target Analysis

The protein-coding differentially expressed genes were searched in the gene database [14] (last accession 20 June 2024) to include the official symbol in humans. Then, the genes were input into the DrugBank database v5.1.10 to search if the differentially expressed genes were previously classified as drug targets [15]. The search usually returned more than one name for the symbol input, thus verifying the coincidence with the UniProt code. The result was reported only when the two coincided; otherwise, it was reported as not the same gene. Some targets were associated with several drugs; only the first ten were considered.

#### 2.2.2. Network Enrichment Analysis

Available information for the relationships between nodes based on Gene Ontology (GO) Biological Process, GO Molecular Function, GO Cellular Components [16], KEGG Ontology [17,18], Reactome [19], UniProt [20], Pfam [21], InterPro [22], Disease Ontology [23], WikiPathways [24,25], and others were included using the STRING network itself [26,27]. The GO terms and pathways were searched in the Comparative Toxicogenomics Database [https://ctdbase.org/ (accessed on 22 June 2024)] to find their association with diseases [28]. The results were sorted by the number of references to report the top one.

#### 2.2.3. The Protein–Protein Interaction Network Analysis

The protein-coding genes were included in the STRING database v12 [26,27]. The STRING database provides a confidence score (from 0 to 1), which estimates the likelihood that an annotated interaction between a pair of proteins is biologically meaningful, specific, and reproducible. In this case, a confidence score of 0.4 or greater was included, selecting all interaction types. The network was analyzed and displayed using the Edge-weighted Spring Embedded Layout with Cytoscape v3.9.1 [29].

## 3. Results

### 3.1. Selection and Characteristics of Primary Articles

**Figure 1:**
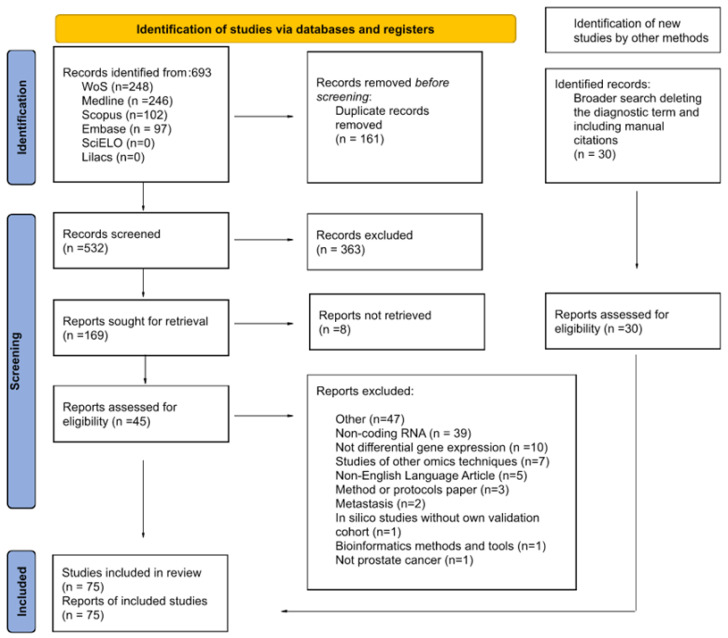
PRISMA flow diagram of the selection process and exclusion criteria of the primary articles in the systematic review found through searches of databases and registers only.

A total of 693 articles were identified up to 24 September 2023 (Figure 1 and Supplementary File, Sheet S1). Of these, 161 duplicate articles were excluded (Supplementary File, Sheet S2), while 363 articles did not meet the eligibility criteria during the screening of titles and abstracts (Supplementary File, Sheet S3). The remaining 169 articles were analyzed by reading the full text, and 124 did not meet the eligibility criteria and were excluded (Supplementary File, Sheet S4). Thirty relevant articles were added from the manual search (Supplementary File, Sheet S5), which raised the final count to 75 articles (Supplementary File, Sheet S6). Most of the selected original articles include a discovery cohort and/or a in silico validation cohort from repositories such as TCGA and GEO, to perform an initial search and selection of differentially expressed genes, as well as a validation cohort to confirm the results obtained previously. The data retrieved in this study, presented below, were extracted from the validation cohort.

In most articles (n = 72), the experimental design was not reported, but all studies include a validation cohort with cases (3253 non-metastatic prostate cancer, mean age 65 ± 14.2 years; range 55–83 years) and controls (n = 2822), which included healthy participants and benign prostatic hyperplasia [BPH] (mean age 64.15 ± 12.9 years; range 40–83 years) as well as adjacent tissue samples (non-cancerous histopathologically verified) from 5003 participants in total. The Gleason scores were ≤6 in 32%, 7 in 31%, and 8–10 in 36% of the PCa cases. The PSA value was significantly higher in cases (average 10.5 ng/mL, min 2.5 ng/mL, and max 43 ng/mL) than in controls (average 5.5 ng/mL, min 1.1 ng/mL, and max 18 ng/mL) (see Table S2 and Supplementary File, Sheet S6). The participants were from Europe (n = 38), Asia (n = 15), North America (n = 15), South America (n = 6), and Oceania (n = 1). Treatments or procedures included radical prostatectomy (n = 46) and simple prostatectomy (n = 9); in 20 articles, this was not reported. The samples include blood (n = 3), urine (n = 9), and tissue (n = 62), and in one article RNA was extracted from tissue and blood. The differential expression analysis (in the validation cohort) was mainly carried out by reverse transcription, with amplification by polymerase chain reaction (RT-qPCR and RT-PCR, n = 71). Various statistical methods were used to assess the differential expression in the articles, including Student’s T method, the Mann–Whitney U test, Chi-square, Fisher’s exact test, and the Kruskal–Wallis test, among others (Supplementary File, Sheet S6).

In the evaluation of methodological quality, the primary articles had a score ranging from 0.40 to 0.95, resulting in a mean score of 0.66 ± 0.12 and a mode of 0.70 (Supplementary File, Sheet S7).

### 3.2. mRNAs Differentially Expressed in Prostate Cancer

A total of 144 mRNAs with significant differential expression (*p* < 0.05, non-metastatic prostate cancer vs. controls [adjacent tissue, healthy or BPH]) were reported in the primary articles (Table S1). The most significant genes were *PSGR*, *WNT5A*, *MacroH2A1.1*, *QKI*, *DDX17*, *ITGBL1*, and *TGM4* (*p*-value < 0.0001). The Fold Change (FC) was not reported in all articles, with the *MMP26* gene being the most upregulated (FC = 30) and *AR* gene the most downregulated (FC = −6). The *AR*, *Erβ*, *PSMA*, *GSTP1*, *hepsin*, *MMP2*, *TGFB1*, and *THBS4* genes were reported on two continents, while *AMACR* was reported as significant in three continents. In addition, *AMACR* was the gene studied in more articles (n = 7), with a total of 385 participants (Supplementary File, Sheet S6). This is followed by *AR*, *TGFB1*, and *PLAU* (also known as *uPA*), reported in three studies, and another 12 genes reported in two studies. The remaining 128 genes were reported only once.

The 144 mRNAs reported were searched in the gene database from NCBI to obtain the official gene symbol. The match between mRNA reported in primary articles and the official name that will be used in subsequent in silico analyses can be found in Supplementary File, Sheet S8. The *TMPRSS2*-*ERG* differential expressed mRNA was discarded in the in silico analysis because it is the product of gene fusion.

### 3.3. Genes Reported as Targets for Drugs Used in Prostate Cancer

The 143 genes were searched in the DrugBank database, identifying 75 genes (proteins) as targets for drugs, and the other 68 genes were not found in the database (Supplementary File, Sheet S9). Of the 75 genes, 36 were targets for drugs used as treatment for various types of cancer, of which 11 were targets for almost 19 drugs related to PCa (Table 1). The KLK3 and FOLH1 proteins were targets for diagnostic and therapeutic agents; the remaining genes were targets for treatment or palliative agents, mostly in approved or investigational status in various types of cancer.

### 3.4. Molecular Pathways Enriched in Prostate Cancer

Of the 143 genes, *VEGFA* was discarded because it does not have an exact match in STRING. The functional enrichment on 142 nodes reported a total of 729 GO Process, 56 GO Function, 37 GO Component terms, and 121 KEGG pathways significantly enriched (FDR < 0.05). The top five GO Process terms were regulation of multicellular organismal process [GO:0051239], response to organic [GO:0010033] and chemical substance [GO:0042221], and positive [GO:0048518] and negative [GO:0048519] regulation of biological process. All of them are related to hypertension and chemical- and drug-induced liver injury. The top five GO function terms were molecular function regulator activity [GO:0098772], ABC-type transporter activity [GO:0140359], protein binding [GO:0005515], endopeptidase activity [GO:0004175], and serine-type endopeptidase activity [GO:0004252], related to cardiomyopathies, hypertension, chemical and drug-induced liver injury, diabetes mellitus, and stroke, respectively. The top five GO component terms were extracellular region [GO:0005576], space [GO:0005615], and exosome [GO:0070062], cell periphery [GO:0071944], and secretory granule [GO:0030141]. These were related to kidney diseases, hypertension, atherosclerosis, breast neoplasms, and inflammation, respectively. All GO terms (or their descendants) were also previously related to prostatic neoplasms, although with fewer supporting references.

The top five KEGG Ontology pathways reported were MicroRNAs in cancer [hsa05206], AGE-RAGE signaling pathway in diabetic complications [hsa04933], Pathways in cancer [hsa05200], Prostate cancer [hsa05215], and ABC transporters [hsa02010]. In this case, the association with the disease is inferred by the number of genes belonging to the group, with breast, prostatic, and colorectal neoplasms being the pathologies that occupy the first places.

Other categories were also analyzed, and 98 diseases, 209 WikiPathways, and 53 Reactome pathways were reported as significantly enriched (see Supplementary File, Sheet S10). The top five diseases reported were Cell type cancer [DOID:0050687], Organ system cancer [DOID:0050686], Cancer [DOID:162], Carcinoma [DOID:305], and Disease of cellular proliferation [DOID:14566], while Prostate cancer [DOID:10283] ranked sixth. In WikiPathways, the top five terms were Malignant pleural mesothelioma [WP5087], Androgen receptor network in prostate cancer [WP2263], Matrix metalloproteinases [WP129], Hepatitis C and hepatocellular carcinoma [WP3646], and Extracellular vesicles in the crosstalk of cardiac cells [WP4300]. Meanwhile, in Reactome, the top five enriched terms were Signal Transduction [HSA-162582], Interleukin-4 and Interleukin-13 signaling [HSA-6785807], Signaling by Interleukins [HSA-449147], Cytokine Signaling in Immune system [HSA-1280215], and ABC-family protein mediated transport [HSA-382556]. All of them, closely related to cancer and prostate cancer, were reported as enriched terms in the first place.

### 3.5. Protein–Protein Interaction Network

**Figure 2:**
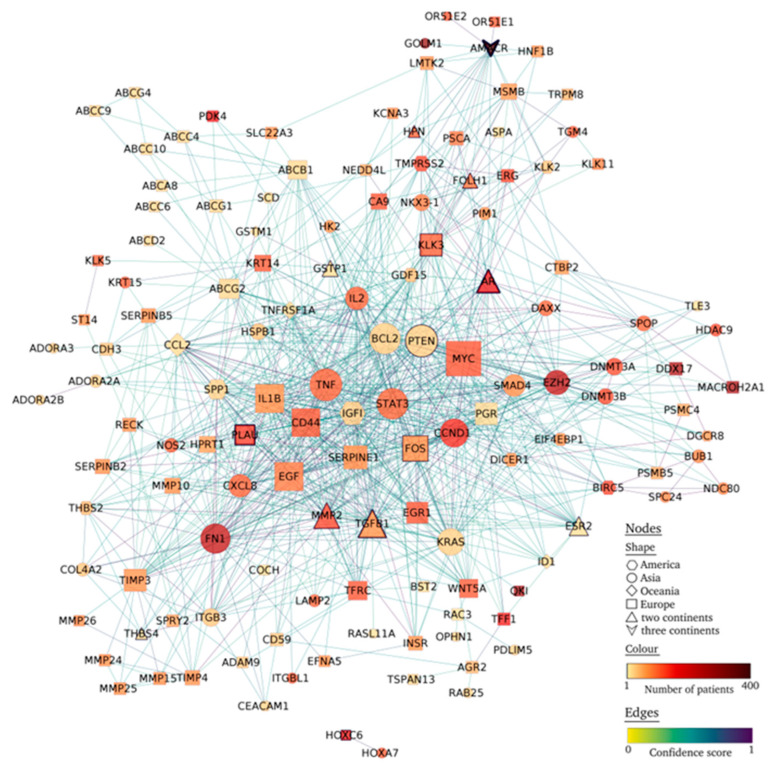
**Protein–protein interaction network of protein-coding genes associated with the diagnosis of prostate cancer.** The network is displayed by Cytoscape and contains 135 nodes and 942 interactions. The size of the nodes is proportional to the number of connections. The node contour increases in proportion to the number of studies reporting it.

In protein–protein interaction network analysis, of the 142 nodes, seven (ZNF154, GCNT1, KLK15, KLK14, TMEM100, RNF19A, RARRES1) were unconnected (confidence score >= 0.04). The resulting network consists of 135 nodes and 942 edges (Figure 2, Supplementary File, Sheets S11 and S12). The top ten more connected nodes (>43 edges) were MYC, PTEN, TNF, STAT3, BCL2, FN1, TGFB1, CCND1, EGF, and IL1B (Figure 2). Of these nodes, the hub node MYC (59 edges) was reported in Europe, the other six nodes were reported in Asian patients, and TGFB1 was reported in two continents (Oceania and Europe). Other highly connected nodes were also reported on two continents, namely MMP2 (39 edges) in Turkey and the United Kingdom, and AR (34 edges) in Turkey, China, and Brazil.

## 4. Discussion

In this study, we summarize all mRNAs reported and validated as differentially expressed in samples from adult patients with prostate cancer. Some of these mRNAs translated protein targets for drugs associated with the treatment of various cancers, including prostate cancer. The molecular pathways enriched were involved in known molecular pathways for cancer, and the hub nodes in the protein network analyses could be used as targets for new treatments or diagnostic strategies for prostate cancer.

Most studies have been conducted in Europe (50.6%), with the largest number of participants (56%), followed by Asia (24.12%). North America is represented only by the USA, with 15 studies, while South America is represented only by Brazil, with six studies. In northern European countries, PCa remains the primary oncological pathology [1]. Europe´s social and economic development is considerably more advanced in comparison to many nations [30]. This allows for greater investment in research activities [31], which could partly explain why the largest number of studies have been reported in this region. However, the sample size of the studies is small, with a range of 7 to 257 participants (average of 73) and a mode in the range of 20 patients; added to the variations in study designs, this limits the possibility of generalizing the findings. This highlights the need to continue investigating these and other mRNAs, with larger validation cohorts spanning more countries and using more standardized study designs.

Regarding the validation method, RT-qPCR was the most frequent analysis technique, which demonstrates that this is a highly sensitive and specific method to detect and measure gene expression in the different stages of the disease [32]. Furthermore, its versatility renders it adaptable to various types of samples, allowing a highly efficient and precise analysis. Additionally, after the COVID-19 pandemic, the RT-qPCR was massively introduced as a clinical diagnostic method, which could facilitate its use as diagnostic strategy [33].

Among the 144 mRNAs summarized, the most reported was alpha-methylacyl-CoA racemase (*AMACR*) gene, which is mentioned in seven primary studies [34,35,36,37,38,39,40] in our systematic review. The *AMACR* gene encodes an enzyme involved in fatty acid metabolism and is identified in specific biological processes such as energy generation, cell proliferation, and apoptosis [41]. Quantitative detection of *AMACR*, based on the RT-qPCR of prostate tissues, may be widely accepted as a tool to monitor patients at risk for PCa [42]. In turn, the gene fusion *TMPRSS2*-*ERG* was reported as differentially expressed in 326 participants from the USA [43,44] and Spain [45]. The relationship of *TMPRSS2*-*ERG* with prostate cancer is well studied in in vivo and in vitro models and has been proposed as a new molecular marker for the diagnosis of this pathology [46]. Another protein-coding gene widely associated with prostate cancer is hepsin (official symbol *HPN*), which was reported as differentially expressed in 33 US patients [47] and 67 patients from the UK [48]. *HPN* encodes a type II transmembrane serine protease associated with the growth and progression of cancers, particularly prostate cancer [49]. It is noteworthy that from 144 mRNAs reported, only 16 have been validated as differentially expressed in more than one primary study that involves samples (cases versus controls) from patients with prostate cancer. This reveals another knowledge gap and highlights the need to validate these findings in a larger number of primary studies using human samples.

However, most of the 144 mRNAs are not specific for prostate cancer, and according to our results, at least 36 of them have already been identified as targets of approved or investigational drugs in different types of cancer. For instance, interleukin 2 (*IL2* [P60568]), reported as differentially expressed in tissue samples from 103 Chinese patients [50], was the target for 12 drugs (most in investigational status), including TG4010 (DB06584). TG4010 is a bivalent cancer vaccine comprised of a modified vaccinia virus Ankara (MVA), with potential immunostimulating and antineoplastic activities and use/treatment in breast cancer, renal cell carcinoma, prostate cancer, and lung cancer [51]. The BCL2 apoptosis regulator [P10415], also validated only in one study that includes 22 patients from Qatar [52], is the target for 14 drugs, with ten of them used for cancer treatment and only two of them for prostate cancer (Table 1). For example, Docetaxel (DB01248) is a clinically well-established anti-mitotic chemotherapy medication, and in combination with cisplatin, is the first-line treatment for prostate cancer [53]. The androgen receptor (*AR* [P10275]), reported in countries of two continents and involving 178 participants [39,50,54], is the target for 83 drugs. For instance, Flutamide (DB00499) is indicated for carcinoma of the prostate, but other drugs such as Fluoxymesterone (DB01185) are also indicated for the treatment of advanced breast cancer [55]. Other targets seem to be specific for prostate cancer, for instance, *PSA* (official symbol *KLK3* [P07288]), *PSMA* (official symbol *FOLH1* [Q04609]), and *PSCA* [O43653]. The gene *KLK3* was reported in 16 patients from Greece [56] and 90 patients from Italy [57]. The gene *FOLH1* was also reported in the Italy study [57] and in 20 patients from Brazil [54]. The gene *PSCA* was reported in 87 patients from Finland [58]. Therefore, there is no relationship between the number of studies that validated an mRNA in patients (or in the number of patients) and the relevance as a therapeutic target that the reported mRNA could have in prostate cancer. In turn, the 68 genes that are not currently targeted by drugs according to DrugBank could be candidates as therapeutic targets for prostate cancer. Furthermore, several of these genes were targets for drug use in the treatment of metastatic PCa, which can indicate that although these genes were deregulated at the beginning of the cancer, they remained deregulated in later stages and even in metastasis.

The function of a protein-coding gene in signaling pathways could be a point of reference for its clinical relevance. In this sense, the main enriched terms in the functional enrichment analysis were widely associated with cancer. The KO hsa05215 (prostate cancer) was the fourth most enriched (FDR = 9.11 × 10^−11^) term. Key protein-coding genes in this group were reported in this study. For instance, Glutathione S-transferases (*GSTP1*) implicating carcinogen defenses [59], growth-factor-signaling pathways (*NKX3.1*, *PTEN*) that regulate the growth and survival of prostate cells [60], and *AR*, which is a transcription factor, are critical determinants of prostate cancer phenotype cells [61]. Also, the androgen receptor network in prostate cancer [WP2263] was the second (FDR = 1.63 × 10^−9^) in WikiPathways, and prostate cancer [DOID:10283] ranked sixth (FDR = 6.76 × 10^−13^) in Disease Ontology. The genes *KLK3*, *PTEN*, *AR*, and *ERG* were common for these categories. The phosphatase and tensin homolog (*PTEN*) gene was reported as differentially expressed in 81 biopsies from Iranian patients [60] and 22 patients from Qatar [52]. The ETS transcription factor (*ERG*) gene was reported in 123 urine samples from US patients [43]. Interestingly, *PTEN* and *ERG* were not reported as cancer drug targets in DrugBank; thus, they may be candidates for therapeutic targeting.

These genes were also highly connected nodes in the network. The hub in the network, MYC proto-oncogene, bHLH transcription factor (*MYC*), was validated only in one study of 114 patients from Germany [62]. The gene *MYC* is a proto-oncogene and encodes a nuclear phosphoprotein that plays a role in cell cycle progression, apoptosis, and cellular transformation. Thus, *MYC* plays a role in various types of cancer [63], not just prostate cancer. The tumor suppressor *PTEN* and the tumor necrosis factor (*TNF*) were the second (degree = 54) and third (degree = 53) more connected nodes, respectively, and like *MYC*, they both play a role in various types of cancer [64]. The *TNF* gene was reported as differentially expressed in 103 tissue samples from Chinese patients [50]. The remaining genes reported as the top ten connected nodes in the protein–protein network analysis were also validated in one or two studies. These genes should be validated in a differentially expressed protein-coding gene diagnostic panel together and not separately. In this regard, five mRNAs (*CCND1*, *LMTK2*, *FN1*, *EZH2*, and *GOLM1*) were included in a gene panel for prostate cancer diagnosis and reported as differentially expressed in a Chinese cohort of 281 patients using RT-qPCR from tissue samples [65]. The authors concluded that multiple biomarkers in combination may provide new tools to detect PCa and distinguish aggressive and indolent PCa for precision and personalized treatment [65]. In another example, Talesa et al. tested five genes with differential expression in urine samples from 90 Italian patients using RT-qPCR, concluding that the diagnostic potential of the combined urinary *PSA* and *PSMA* level was significantly better than that of each singularly considered marker (including the gold standard total serum PSA) [57].

Based on the in silico results of the 144 mRNAs summarized here, whose differential expression has already been validated in patients, we propose a protein-coding gene expression profile composed of *MYC*, *PTEN*, *TNF*, *STAT3*, *BCL2*, *FN1*, *TGFB1*, *CCND1*, *EGF*, *IL1B*, *CD44*, *KRAS*, *KLK3*, *PTEN*, *AR*, *ERG*, *AMACR*, *IL2*, *PGR*, *HSPB1*, *FOLH1*, *BIRC5*, *PSCA*, *SLC22A3*, and *ABCC10*, to be used in the clinical diagnosis of prostate cancer.

### 4.1. Limitation

The lack of information in certain articles related to the methodological design made it difficult to compare and synthesize the studies included in this review.

In addition, the limited sample size prevented confirmation of statistical correlations between mRNAs and PCa. To address this issue, it is vital to increase the methodological soundness of primary articles, especially case–control studies, and expand the number of participants. Evaluation of the articles was a challenge in light of the lack of adequate instruments to measure their quality. The existing assessment instruments focused primarily on preclinical and clinical practice studies, rather than the essential elements of this analysis. A generic instrument for quantitative studies was used in this study; there were limitations when evaluating certain factors such as blinding of participants and researchers, sample size calculation, and control of confounding variables.

### 4.2. Systematic Review Biases

The lack of information on some variables could affect both the internal validity and the robustness of the results. To minimize the publication and selection bias inherent in this design, all publications related to the topic were included, assigning articles randomly and masking when selecting the studies [66]. All articles included in this systematic review involved a control group, which meant that their mRNAs were validated in terms of their use and their relationship with the diagnosis, therapy, follow-up, and prognosis of PCa.

## 5. Conclusions

Based on our in silico findings on the 144 mRNAs summarized here, whose significant differential expression has already been validated in patients, we propose a protein-coding gene expression profile of twenty-five mRNAs with the potential to be used in the clinical diagnosis of prostate cancer, using a larger validation cohort and appropriate study design.

## Figures and Tables

**Table 1:** **Genes classified as drug targets in the DrugBank database.** Protein-coding genes as targets for drugs related to the treatment of prostate cancer in all states (investigation, experimental, approved). In the case of target genes for many drugs, only the first 10 are reported.

Drug ID	Name	Short Indication * [Status]	Target [UniProt ID]
DB01248	Docetaxel	A taxoid antineoplastic agent used in the treatment of various cancers, including metastatic prostate cancer [approved, investigation].	BCL2 [P10415]
DB05297	Paclitaxel docosahexaenoic acid	A type of mitotic inhibitor in cancers including prostate [investigation].	
DB00499	Flutamide	An antiandrogen used for locally confined stage B2-C and D-2 metastatic prostate carcinoma [approved, investigation].	AR [P10275]
DB01128	Bicalutamide	An androgen receptor inhibitor used to treat Stage D2 metastatic carcinoma of the prostate [approved].	
DB00665	Nilutamide	An antineoplastic hormone used to treat prostate cancer [approved, investigation].	
DB06584	TG4010	Investigated for use/treatment in breast cancer, renal cell carcinoma, prostate cancer, and lung cancer [investigation].	IL2 [P60568]
DB04839	Cyproterone acetate	Used alone at higher doses for palliative treatment of patients with prostate cancer [approved, investigation].	KLK3 [P07288]
DB16019	Gallium Ga-68 gozetotide	A radioactive diagnostic agent indicated for positron emission tomography (PET) of prostate-specific membrane antigen (PSMA)-positive lesions in men with prostate cancer metastasis [approved].	
DB16778	Lutetium Lu-177 vipivotide tetraxetan	A radioligand therapeutic agent used to treat prostate-specific membrane antigen (PSMA)-positive metastatic castration-resistant prostate cancer in adults [approved].	
DB02998	Metribolone	A synthetic non-aromatizable androgen and anabolic steroid. It binds strongly to the androgen receptor and has therefore also been used as an affinity label for this receptor in the prostate and prostatic tumors [experimental].	
DB00351	Megestrol acetate	Palliative management of recurrent, inoperable, or metastatic breast cancer, endometrial cancer, and prostate cancer [approved, investigational].	PGR [P06401]
DB06094	Apatorsen	A second-generation antisense drug that in preclinical experiments inhibits the production of Heat Shock Protein 27 (Hsp27), a cell survival protein found at elevated levels in many human cancers, including prostate, lung, breast, ovarian, bladder, renal, pancreatic, multiple myeloma, and liver cancer [investigational].	HSPB1 [P04792]
DB00089	Capromab pendetide	A monoclonal anti-prostate specific membrane antigen antibody used in imaging kits to target radioactive agents to malignant prostate tissue [approved, investigational].	FOLH1 [Q04609]
DB14805	Piflufolastat F 18	Radiopharmaceutical diagnostic agents used with PET to image PSMA-positive lesions for the diagnosis of metastatic or recurrent prostate cancer [approved, investigational].	
DB17851	Flotufolastat F-18		
DB05141	LY2181308	An antisense oligonucleotide that potently downregulates survivin expression in human cancer cells derived from lung, colon, breast, prostate, ovary, cervix, skin, and brain [investigational].	BIRC5 [O15392]
DB05933	MK-4721	A fully human monoclonal antibody directed to Agensys’ proprietary target Prostate Stem Cell Antigen (PSCA), an antigen expressed at significant levels on tumor cells from the majority of patients with all stages of prostate, pancreatic, and bladder cancers [investigation].	PSCA [O43653]
DB00783	Estradiol	An estrogenic steroid used to treat (among others) advanced androgen-dependent carcinoma of the prostate [approved, investigational].	SLC22A3 [O75751], ABCC10 [Q5T3U5]

* Full description and/or indication and/or summary in Supplementary File, Sheet S9.

## Data Availability

The original contributions presented in the study are included in the article/Supplementary Materials, further inquiries can be directed to the corresponding author.

## Supplementary Materials

The following supporting information can be downloaded at: https://www.mdpi.com/article/10.3390/biomedicines12112509/s1, Table S1: Summary of mRNAs with significant differential expression in patients with prostate cancer when comparing cases vs controls. Data retrieved from primary articles, including all references of the 75 primary articles analyzed; Table S2: PCa and controls PSA values, reported in data retrieved from primary articles; Supplementary Excel file: Systematic review of the literature and in silico analysis, step by step, with all results to reproduce the article.
