# Supplementary material for: Differential Protein-Coding Gene Expression Profile in Patients with Prostate Cancer
Source: Biomedicines. 2024 Nov 1;12(11):2509. doi: 10.3390/biomedicines12112509 (PMC11592252; doi:10.3390/biomedicines12112509)
Supplement: Supplementary file 1 [file biomedicines-12-02509-s001.zip › Supplementary_Tables.pdf]

Table S1. Summary of mRNAs with significant differential expression in patients with prostate cancer when comparing cases vs controls. Data retrieved from primary articles.

Publication	Record	Target Symbol	Descrption	Value	Fold Change
Demidenko R., et al.	(1)	ABCA8; ABCB1; ABCC6; ABCC9; ABCC10; ABCD2; ABCG2; ABCG4; ABCC4; ABCG1.	ATP-binding cassette (ABC) transporters are transmembrane proteins; Subfamily A member8; Subfamily B member1; Subfamily C member6; Subfamily C member9; Subfamily C member10; Subfamily D member2; Subfamily G member2; Subfamily G member4; Subfamily C member4; Subfamily G member1.	0.029; 0.035; 0.005; 0.001; 0.030; 0.001; 0.003; 0.001; 0.007; 0.029.	2.03; 1.52; 5.67; 2.77; 1.24; 2.44; 2.22; 2.14; 2.49; 1.48.
Fritzche F., et al.	(2)	ADAM9	A disintegrin and metalloprotease	0.0002	not reported
Mousavi S., et al.	(3)	ADORA2A; ADORA2B; ADORA3.	Adenosine A2a receptor; Adenosine A2b receptor; Adenosine A3 receptor.	0.05; 0.001; 0.05.	1.5; 2.4; 1.6.
Neen A., et al.	(4)	AGR2	Anterior gradient 2	0.0001	not reported
Ouyang B., et al.	(5)	AMACR	Alpha-methylacyl-CoA racemase	0.05	not reported
Ouyang B., et al	(6)	AMACR	Alpha-methylacyl-CoA racemase	0.006	not reported
Eryilmaz I., et al.	(7)	AMACR	Alpha-methylacyl-CoA racemase	0.034	not reported
Schostak M., et al.	(8)	AMACR	Alpha-methylacyl-CoA racemase	0.0001	3.75
Rubin M., et al.	(9)	AMACR	Alpha-methylacyl-CoA racemase	0.05	not reported
Erylmarz I., et al.	(10)	AMACR; AR; MMP2.	Alpha-methylacyl-CoA racemase; Androgen receptor; Matrix metallopeptidase 2.	0.016; 0.016; 0.013.	≤6.31; ≤6.26; ≤6.81.
Moore S., et al.	(11)	AMACR; GSTP1; SCD.	Alpha-methylacyl-CoA racemase; Glutathione-S-transferase; Stearoyl-CoA desaturase.	0.001	AMACR: 2-4 fold; GSTP1: since -1.5 fold to 1 fold; SCD: since -2 fold to 2 fold.
Alonso V. et al	(12)	AR; CEACAM-1; OPN-1; PSMA.	Androgen receptor; Carcinoembryonic antigen-related cell adhesion molecule; Osteopontin 1; Prostate-specific membrane antigen.	0.05	1.50; 1.65; 1.51; 1.64.
Huang T., et al.	(13)	AR; IL-2; IL-8; iNOS; TNF-α; VEGF;	Androgen receptor; Interleukin 2; Interleukin 8; Inducible nitric oxide synthase; Tumor necrosis factor-alpha; Vascular endothelial growth factor.	0.006; 0.009; 0.003; 0.003; 0.002; 0.0001;	not reported
van der Heul-Nieuwenhuijsen L,. et al.	(14)	ASPA; BST2; COCH; FLJ10970; THBS4	Aspartoacylase; Bone marrow stromal cell antigen 2; coagulation factor C homologue; the hypothetical protein	0.01	not reported

			FLJ10970; Thrombospondin 4.		
Shan J., et al.	(15)	BCL2; KRAS; PTEN.	B cell leukemia/lymphoma 2; Kirsten rat sarcoma viral oncogene homolog; Phosphatase and tensin homolog.	0.006; 0.007; 0.010.	not reported
Motamedinia P., et al.	(16)	BIRC5; ERG; TMPRSS2:ERG; TMPRSS2; TMPRSS2:ERG in tissue.	Baculoviral IAP repeat containing 5; ETS transcription factor; Transmembrane protease serine 2; Transmembrane protease serine 2.	0.004; 0.003; 0.013; 0.019; 0.009.	not reported; TMPRSS2:ERG in tissue 98 fold
Chen S., et al.	(17)	BUB1; NDC80; SPC24.	Mitotic Checkpoint Serine/Threonine Kinase; NDC80 Kinetochore Complex Component; SPC24 Component Of NDC80 Kinetochore Complex.	0.05	not reported
Malentacchi F., et al	(18)	CAIX	Carbonic anhydrase IX	0.001	not reported
Xiao K., et al.	(19)	CCND1; EZH2; FN1; GOLM1; LMTK2.	Cell cycle regulator cyclin D1; Enhancer of zeste 2 polycomb repressive complex 2 subunit; Fibronectin; Golgi membrane protein 1; Lemur tyrosine kinase 2.	0.0001	not reported
Carneiro I., et al.	(20)	CD44; KRT14; WNT5A.	Cluster of differentiation 44; Keratin 14; Wingless-type MMTV integration site family member 5A.	0.013; 0.004; 0.001.	not reported
Xu C. et al.	(21)	CD59	Human Protectin	0.0295	not reported
Royo F., et al.	(22)	CDH3	Cadherin 3 type 1 gene	0.018	not reported
Zhu J., et al.	(23)	COL4A2; HSPB1; ITGB3;	Collagen Type IV Alpha 2 Chain; Heat shock protein beta-1; Integrin Subunit Beta 3.	0.05	>0.2; >0.13; >0.64.
Harries L. et al.	(24)	CTBP2; HNF1B; LMTK2; MSMB; SLC22A3;	C-terminal binding protein 2;HNF homeobox 1 beta; Lemur tyrosine kinase 2; Microseminoprotein beta; Solute carrier family 22 extraneuronal monoamine transporter member 3.	0.008; 2.9x10-9; 2.4 × 10-7; 4.0x10-10; 0.015.	not reported
Fuessel S., et al.	(25)	D-GPCR	Dresden G protein-coupled receptor	0.0006	>3
Jamali L., et al.	(26)	DAXX; LAMP2; RARRES1; SPOP.	Deathdomain associated protein; Lysosome-associated membrane glycoprotein 2; Retinoic acid receptor responder 1; Speckle-type POZ protein.	DAXX (p=0.001),LAMP 2 (p=0.001), RARRES1 (Normal P=0.001, BPH p=0.011), SPOP (P=0.001).	not reported
Vieira T., et al.	(27)	DDX17; MacroH2A1.1; QKI.		0.0001	2.11; 1.33; 1.65.

Ambs S., et al.	(28)	DICER; DGCR8.	Cytosolic RNase III enzyme; GCR8 microprocessor complex subunit.	0.001	not reported
Mohammadi M., et al.	(29)	DNMT3A; DNMT3B; GSTP1; HDAC.	DNA methyltransferases (DN22 MTs) from which DNMT3A and DNMT3B; Histone deacetylases; Glutathione S-transferase pi 1;	DNMT3A (N: p=0.005, BPH p=0.0002), DNMT3B (N: p=0.04, BPH p=0.02); HDAC (N: 0.0001, BPH p=0.04); GSTP1 (N:p=0.0001, BPH: 0.002).	not reported
Rosenberg E., et al.	(30)	EFNA5; FOS; IL1B; PLAU; TGFB1.	Ephrin A5; Fos proto-oncogene; AP-1 transcription factor subunit; Interleukin 1 beta; Plasminogen activator urokinase; Transforming growth factor beta 1.	0.0584; 0.0575; 0.0866; 0.038; 0.0056.	>2-fold
Soulitzis N., et al.	(31)	EGF; TGFB1; VEGF;	Epidermal growth factor; Transforming growth factor beta 1; Vascular endothelial growth fator.	0.003; 0.002; 0.008.	not reported
Eid M., et al.	(32)	EGR-1	Early growth response 1	0.001	not reported
Schlomm T., et al.	(33)	EGR1; FOLH1; FOS; MYC; TFRC.	Early growth response 1; Folate hydrolase (prostate-specific membrane antigen) 1; V-fos FBJ murine osteosarcoma viral oncogene homolog; V-myc myelocytomatosis viral oncogene homolog (avian); Transferrin receptor.	0.002	0.52; 1.93; 0.30; 1.67; 1.87.
Ozen M., et al.	(34)	EIF4EBP1	Eukaryotic translation initiation factor 4E binding protein 1	0.01	not reported
Abd Elmageed Z., et al	(35)	ER β	Estrogen receptor beta	0.01	> 3
Walton T., et al.	(36)	Erβ; PGR.	Estrogen receptor beta; Progesterone receptor.	0.01; 0.05.	Erβ > 4.5
Chen Z., et al.	(37)	GCNT1	Glycosyltransferase	0.001	not reported
Kang H., et al.	(38)	GDF-15; PDLIM5; THBS4.	Growth Differencetion Factor 15; PDZ And LIM Domain 5; Trombospondina 4.	0.0001	>4
Chetcuti A., et al.	(39)	GSTM1; ID-1; MCP-1; TGF-B1; TNFR1.	Glutathione S-transferase M1; Inhibitor of DNA binding-1; Monocyte chemotactic protein-1; Transforming growth factor beta1; Tumor necrosis factor-α receptor-1.	0.05	<3.00; <4.07; <3.71; <3.37; <3.04;
Riddick A., et al.	(40)	Hepsin; Maspin; MMP2; MMP10; MMP15; MMP23; MMP24; MMP25; MMP26;	Hepsin; Maspin; Matrix metallopeptidase 2; 10; 15; 23; 24; 25; 26; Matriptase-1; Plasminogen activator inhibitor-1; Plasminogen activator inhibitor-2; Reversion inducing	0,0001; 0,001; 0.004; 0.016; 0,0001; 0,0001; 0,014; 0,003; 0,0001; 0,0001; 0,001; 0,032; 0,0001; 0,001; 0,004; 0,04.	MMP-26 >30-fold

		MTSP1; PAI1; PAI2; RECK; TIMP3; TIMP4; uPA;	cysteine rich protein with kazal motifs; Matrix metallopeptidase inhibitor 3; 4; Urokinase-type plasminogen activator.		
Welsh J., et al.	(41)	Hepsin.; MIC-1.	Hepsin.; Secreted macrophage inhibitory cytokine.	0.05	>5
He H., et al.	(42)	hK2; PIM-1.	Human glandular kallikrein; Serine-threonine kinase.	CP vs normal. hK2 p=0,0068 y PIM-1 p=0,0071, CP vs BPH. hK2 p=0.0127 y PIM-1 p=0,023	not reported
Shan M., et al.	(43)	HOXA7; ITGBL1; KRT15; TGM4.	Homeobox A7; Integrin beta-like 1; Keratin 15; Transglutaminasa 4.	0.0001; 0.006; 0.005; 0.0001.	not reported
Yazbek Hanna M., et al.	(44)	HOXC6	Homeobox C6	0.022	>2-3
Mengual L., et al.	(45)	HOXC6; PDK4; TMPRSS2-ERG.	Homeoblox C6; Pyruvate dehydrogenase kinase 4; Transmembrane protease serine 2: ETS transcription factor.	0.05	>1.5
Mahn R., et al	(46)	HPRT1	Messenger RNA, hypoxanthine phosphoribosyltransferase 1	0.05	not reported
Figueroa J. et al	(47)	IGF-I	Insulin-like growth factor 1	0.014	not reported
Heni M., et al.	(48)	INSR transcript variant X2/transcript variant X1	Insulin receptor substrate (IRS)-1 and IRS-2	0.05	not reported
Nakamura T., et al.	(49)	KLK11	Hippostasin/kallikrein 11	0.056	not reported
Yousef G., et al	(50)	KLK14	Kallikrein related peptidase 14	0.001	2.5
Mavridis K., et al	(51)	KLK15	Kallikrein related peptidase 15	0.001	7.4
Meola J., et al.	(52)	KLK2	Kallikrein related peptidase 2	0.0001	not reported
(58) Korbakis D., et al.	(53)	KLK5	Kallikrein related peptidase 5	0.001	not reported
Hellwinkel OJC., et al.	(54)	NEDD4L; PSMC4; PSMB5.	Neural precursor cell expressed developmentally downregulated 4-like; Proteasome (prosome macropain) 26S subunit ATPase 4; Proteasome (prosome macropain) subunit b type 5.	0.011; 0.027; 0.001.	2.75; 1.57; 2.
Nodouzi V., et al.	(55)	NKX3.1; PTEN	NK3 Homeobox 1; Phosphatase and tensin homolog.	0.004; 0.001.	< 0.155; <0.003
Tilli T., et al.	(56)	OPN	Osteopontin	0.01	3-4
Colombel M., et al.	(57)	PS2	Trefoil protein	0.001	not reported
Pampalakis G., et al.	(58)	PSA-SV5	Splice variant of prostate-specific antigen	0.036	not reported
Talesa V., et al.	(59)	PSA; PSMA.	PCa antigen 3; Encoding for prostate-specific membrane antigen.	0.0001; 0.016.	not reported

Väänänen R., et al.	(60)	PSCA	Prostate stem cell antigen	0.046	>2.9
Xu LL., et al.	(61)	PSGR	G-protein-coupled olfactory receptor family	0.0001	>1.5
Hu., et al.	(62)	Rab25	Ras-related protein 25	0.001	not reported
Engers R., et al.	(63)	Rac3	Rac family small GTPase 3 ubiquitous Rac3	0.018	3.10
Louro R., et al.	(64)	RASL11A	RAS-like, family 11, member A	0.00065	2.3
Bai V., et al.	(65)	RNF19A	The Ring finger protein 19A	0.0066	>2
Zhang D., et al.	(66)	Smad4	SMAD family member 4	0.001	not reported
Fritzsche S., et al.	(67)	SPRY2	Sprouty RTK signaling antagonist 2	0.017	2
Ahani M., et al.	(68)	STAT3	Signal transducer and activator of transcription 3	0.001	>3
Nakaya H., et al.	(69)	TLE3	TLE family member 3 transcrptional corepressor	0.01	6-17
Salami S., et al	(70)	TMPRSS2-ERG	Transmembrane protease serine 2: ETS transcription factor	0.001	not reported
Fuessel S., et al.	(71)	trp-p8	Transient receptor potential p8	0.0048	not reported
Matos A., et al.	(72)	TSP2	Thrombospondin 2	0.005	not reported
Arencibia J., et al.	(73)	TSPAN13	Tetraspanin-13	0.05	>2
Janabi O., et al.	(74)	uPA	Urokinase-type plasminogen activator	0.004	not reported
Zhang W., et al.	(75)	ZNF154	Zinc finger protein 154	0.004	not reported

Table S2. PCa and controls PSA values, reported in data retrieved from primary articles.

PCa		Controls	
PSA (ng/ml)	Number of patients	PSA (ng/ml)	Number of participants
0 to < 4	159	0 to < 4	76
4 to < 10	744	4 to < 10	55
10 to 20	490	10 to 20	5
> 20	107	> 20	1
Unkonowed	24	Unkonowed	2

1. Demidenko R, Razanauskas D, Daniunaite K, Lazutka JR, Jankevicius F, Jarmalaite S. Frequent down-regulation of ABC transporter genes in prostate cancer. BMC Cancer [Internet]. 2015 Oct 12 [cited 2023 Aug 24];15(1). Available from: /pmc/articles/PMC4603841/
2. Fritzsche FR, Jung M, Tölle A, Wild P, Hartmann A, Wassermann K, et al. ADAM9 Expression is a Significant and Independent Prognostic Marker of PSA Relapse in Prostate Cancer. Eur Urol. 2008 Nov 1;54(5):1097–108.
3. Mousavi S, Panjehpour M, Izadpanahi MH, Aghaei M. Expression of adenosine receptor subclasses in malignant and adjacent normal human prostate tissues. Prostate [Internet]. 2015 May 1 [cited 2024 Jun 18];75(7):735–47. Available from: https://onlinelibrary.wiley.com/doi/full/10.1002/pros.22955
4. Neeb A, Hefele S, Bormann S, Parson W, Adams F, Wolf P, et al. Splice variant transcripts of the anterior gradient 2 gene as a marker of prostate cancer. Oncotarget [Internet]. 2014 [cited 2024 Jun 18];5(18):8681. Available from: /pmc/articles/PMC4226713/
5. Ouyang B, Leung YK, Wang V, Chung E, Levin L, Bracken B, et al. α-Methylacyl-CoA Racemase Spliced Variants and Their Expression in Normal and Malignant Prostate Tissues. Urology. 2011 Jan 1;77(1):249.e1-249.e7.

6. Ouyang B, Bracken B, Burke B, Chung E, Liang J, Ho SM. A Duplex qPCR Assay Based on Quantification of α-Methylacyl-CoA Racemase transcripts and Prostate Cancer Antigen 3 in Urine Sediments Improved Diagnostic Accuracy for Prostate Cancer. J Urol [Internet]. 2009 Jun [cited 2024 Jun 18];181(6):2508. Available from: /pmc/articles/PMC4372725/
7. Eryilmaz IE, Kordan Y, Vuruskan BA, Kaygısız O, Tunca B, Cecener G. T2E (TMPRSS2-ERG) fusion transcripts are associated with higher levels of AMACR mRNA and a subsequent prostate cancer diagnosis in patients with atypical small acinar proliferation. Gene. 2018 Mar 1;645:69–75.
8. Schostak M, Miller K, Krause H, Schrader M, Kempkensteffen C, Kollermann J. Kinetic fluorescence reverse transcriptase-polymerase chain reaction for alpha-methylacyl CoA racemase distinguishes prostate cancer from benign lesions. Cancer Detect Prev. 2006 Jan 1;30(5):449–54.
9. Rubin MA, Zhou M, Dhanasekaran SM, Varambally S, Barrette TR, Sanda MG, et al. α-Methylacyl Coenzyme A Racemase as a Tissue Biomarker for Prostate Cancer. JAMA [Internet]. 2002 Apr 3 [cited 2023 Aug 24];287(13):1662–70. Available from: https://jamanetwork.com/journals/jama/fullarticle/194790
10. Eryilmaz IE, Aytac Vuruskan B, Kaygısız O, Egeli U, Tunca B, Kordan Y, et al. RNA-based markers in biopsy cores with atypical small acinar proliferation: Predictive effect of T2E fusion positivity and MMP-2 upregulation for a subsequent prostate cancer diagnosis. Prostate [Internet]. 2019 Feb 1 [cited 2024 Jun 18];79(2):195–205. Available from: https://onlinelibrary.wiley.com/doi/full/10.1002/pros.23724
11. Moore S, Knudsen B, True LD, Hawley S, Etzioni R, Wade C, et al. Loss of stearoyl-CoA desaturase expression is a frequent event in prostate carcinoma. Int J Cancer. 2005 Apr 20;114(4):563–71.
12. Alonso V, Neves AF, Marangoni K, De Faria PCB, Cordeiro ER, Freschi APP, et al. Gene Expression Profile of Prostate Cancer Patients by Chemiluminescent Analysis. http://dx.doi.org/101080/00032710802568655 [Internet]. 2009 Jan [cited 2023 Aug 24];42(1):166–77. Available from: https://www.tandfonline.com/doi/abs/10.1080/00032710802568655
13. Huang TR, Wang GC, Zhang HM, Peng B. Differential research of inflammatory and related mediators in BPH, histological prostatitis and PCa. Andrologia [Internet]. 2018 May 1 [cited 2023 Aug 24];50(4):e12974. Available from: https://onlinelibrary.wiley.com/doi/full/10.1111/and.12974
14. Van Der Heul-Nieuwenhuijsen L, Hendriksen PJM, Van Der Kwast TH, Jenster G. Gene expression profiling of the human prostate zones. BJU Int [Internet]. 2006 Oct 1 [cited 2023 Aug 24];98(4):886–97. Available from: https://onlinelibrary.wiley.com/doi/full/10.1111/j.1464-410X.2006.06427.x
15. Shan J, Al-Rumaihi K, Chouchane K, Al-Bozom I, Rabah D, Farhat K, et al. Prostate cancer small non-coding RNA transcriptome in Arabs. J Transl Med [Internet]. 2017 Dec 21 [cited 2023 Aug 24];15(1):260. Available from: /pmc/articles/PMC5740966/
16. Motamedinia P, Scott AN, Bate KL, Sadeghi N, Salazar G, Shapiro E, et al. Urine Exosomes for Non-Invasive Assessment of Gene Expression and Mutations of Prostate Cancer. PLoS One [Internet]. 2016 May 1 [cited 2024 Jun 18];11(5):e0154507. Available from: https://journals.plos.org/plosone/article?id=10.1371/journal.pone.0154507
17. Chen S, Wang X, Zheng S, Li H, Qin S, Liu J, et al. Increased SPC24 in prostatic diseases and diagnostic value of SPC24 and its interacting partners in prostate cancer. Exp Ther Med [Internet]. 2021 Jun 30 [cited 2024 Jun 18];22(3). Available from: /pmc/articles/PMC8281004/
18. Malentacchi F, Vinci S, Melina A Della, Kuncova J, Villari D, Nesi G, et al. Urinary carbonic anhydrase IX splicing messenger RNA variants in urogenital cancers. Urol Oncol Semin Orig Investig. 2016 Jul 1;34(7):292.e9-292.e16.

19. Xiao K, Guo J, Zhang X, Feng X, Zhang H, Cheng Z, et al. Use of two gene panels for prostate cancer diagnosis and patient risk stratification. Tumor Biol [Internet]. 2016 Aug 1 [cited 2023 Aug 26];37(8):10115–22. Available from: https://link.springer.com/article/10.1007/s13277-015-4619-0
20. Carneiro I, Quintela-Vieira F, Lobo J, Moreira-Barbosa C, Menezes FD, Martins AT, et al. Expression of EMT-Related Genes CAMK2N1 and WNT5A is increased in Locally Invasive and Metastatic Prostate Cancer. J Cancer [Internet]. 2019 [cited 2023 Aug 24];10(24):5915. Available from: /pmc/articles/PMC6856586/
21. Xu C, Jung M, Burkhardt M, Stephan C, Schnorr D, Loening S, et al. Increased CD59 protein expression predicts a PSA relapse in patients after radical prostatectomy. Prostate [Internet]. 2005 Feb 15 [cited 2023 Aug 24];62(3):224–32. Available from: https://onlinelibrary.wiley.com/doi/full/10.1002/pros.20134
22. Royo F, Zuñiga-Garcia P, Torrano V, Loizaga A, Sanchez-Mosquera P, Ugalde-Olano A, et al. Transcriptomic profiling of urine extracellular vesicles reveals alterations of CDH3 in prostate cancer. Oncotarget [Internet]. 2016 Feb 2 [cited 2024 Jun 18];7(6):6835. Available from: /pmc/articles/PMC4872752/
23. Zhu J, Pan C, Jiang J, Deng M, Gao H, Men B, et al. Six stroma-based RNA markers diagnostic for prostate cancer in European-Americans validated at the RNA and protein levels in patients in China. Oncotarget [Internet]. 2015 Jun 6 [cited 2023 Aug 24];6(18):16757. Available from: /pmc/articles/PMC4599305/
24. Harries LW, Perry JRB, McCullagh P, Crundwell M. Alterations in LMTK2, MSMB and HNF1B gene expression are associated with the development of prostate cancer. BMC Cancer [Internet]. 2010 Jun 22 [cited 2023 Aug 24];10:315. Available from: /pmc/articles/PMC2908099/
25. Fuessel S, Weigle B, Schmidt U, Baretton G, Koch R, Bachmann M, et al. Transcript quantification of Dresden G protein-coupled receptor (D-GPCR) in primary prostate cancer tissue pairs. Cancer Lett. 2006 May 8;236(1):95–104.
26. Jamali L, Moradi A, Ganji M, Ayati M, Kazeminezhad B, Attar ZF, et al. Potential Prognostic Role for SPOP, DAXX, RARRES1, and LAMP2 as an Autophagy Related Genes in Prostate Cancer. Urol J [Internet]. 2020 Mar 16 [cited 2023 Aug 24];17(2):156–63. Available from: https://journals.sbmu.ac.ir/urolj/index.php/uj/article/view/4935
27. Vieira-Silva TS, Monteiro-Reis S, Barros-Silva D, Ramalho-Carvalho J, Graça I, Carneiro I, et al. Histone variant MacroH2A1 is downregulated in prostate cancer and influences malignant cell phenotype. Cancer Cell Int [Internet]. 2019 Apr 29 [cited 2024 Jun 18];19(1):1–13. Available from: https://cancerci.biomedcentral.com/articles/10.1186/s12935-019-0835-9
28. Ambs S, Prueitt RL, Yi M, Hudson RS, Howe TM, Petrocca F, et al. Genomic profiling of microRNA and mRNA reveals deregulated microRNA expression in prostate cancer. Cancer Res [Internet]. 2008 Aug 8 [cited 2023 Aug 24];68(15):6162. Available from: /pmc/articles/PMC2597340/
29. Mohammadi M, Irani S, Salahshourifar I, Hosseini J, Moradi A, Pouresmaeili F. Investigation of GSTP1 and epigenetic regulators expression pattern in a population of Iranian patients with prostate cancer. Hum Antibodies. 2020 Jan 1;28(4):327–34.
30. Expression of cancer-associated genes in prostate tumors | Experimental oncology [Internet]. [cited 2023 Aug 24]. Available from: https://exp-oncology.com.ua/article/9723
31. Soulitzis N, Karyotis I, Delakas D, Spandidos DA. Expression analysis of peptide growth factors VEGF, FGF2, TGFB1, EGF and IGF1 in prostate cancer and benign prostatic hyperplasia. Int J Oncol [Internet]. 2006 Aug 1 [cited 2024 Jun 18];29(2):305–14. Available from: http://www.spandidos-publications.com/10.3892/ijo.29.2.305/abstract
32. Eid MA, Kumar MV, Iczkowski KA, Bostwick DG, Tindall DJ. Expression of Early

Growth Response Genes in Human Prostate Cancer1. CANCER Res [Internet]. 1998 [cited 2023 Aug 26];58:246–246. Available from: http://aacrjournals.org/cancerres/article-pdf/58/11/2461/2466568/cr0580112461.pdf
33. Schlomm T, Hellwinkel OJC, Buness A, Ruschhaupt M, Lübke AM, Chun FK, et al. Molecular Cancer Phenotype in Normal Prostate Tissue. Eur Urol. 2009 Apr 1;55(4):885–91.
34. Ozen M, Creighton CJ, Ozdemir M, Ittmann M. Widespread deregulation of microRNA expression in human prostate cancer. Oncogene 2008 2712 [Internet]. 2007 Sep 24 [cited 2023 Aug 24];27(12):1788–93. Available from: https://www.nature.com/articles/1210809
35. Abd Elmageed ZY, Moroz K, Srivastav SK, Fang Z, Crawford BE, Moparty K, et al. High circulating estrogens and selective expression of ERβ in prostate tumors of Americans: implications for racial disparity of prostate cancer. Carcinogenesis [Internet]. 2013 Sep 1 [cited 2024 Jun 18];34(9):2017–23. Available from: https://dx.doi.org/10.1093/carcin/bgt156
36. Walton TJ, Li G, McCulloch TA, Seth R, Powe DG, Bishop MC, et al. Quantitative RT-PCR analysis of estrogen receptor gene expression in laser microdissected prostate cancer tissue. Prostate [Internet]. 2009 Jun 1 [cited 2023 Aug 24];69(8):810–9. Available from: https://onlinelibrary.wiley.com/doi/full/10.1002/pros.20929
37. Chen Z, Gulzar ZG, St. Hill CA, Walcheck B, Brooks JD. Increased Expression of GCNT1 is Associated With Altered O-glycosylation of PSA, PAP, and MUC1 in Human Prostate Cancers. Prostate [Internet]. 2014 [cited 2023 Aug 24];74(10):1059. Available from: /pmc/articles/PMC5862140/
38. Kang HW, Lee HY, Byun YJ, Jeong P, Yoon JS, Kim DH, et al. A novel urinary mRNA signature using the droplet digital polymerase chain reaction platform improves discrimination between prostate cancer and benign prostatic hyperplasia within the prostate-specific antigen gray zone. Investig Clin Urol [Internet]. 2020 Jul 1 [cited 2024 Jun 18];61(4):411–8. Available from: https://doi.org/10.4111/icu.2020.61.4.411
39. Chetcuti A, Margan S, Mann S, Russell P, Handelsman D, Rogers J, et al. Identification of differentially expressed genes in organ-confined prostate cancer by gene expression array. Prostate [Internet]. 2001 May 1 [cited 2023 Aug 26];47(2):132–40. Available from: https://onlinelibrary.wiley.com/doi/full/10.1002/pros.1056
40. Riddick ACP, Shukla CJ, Pennington CJ, Bass R, Nuttall RK, Hogan A, et al. Identification of degradome components associated with prostate cancer progression by expression analysis of human prostatic tissues. Br J Cancer [Internet]. 2005 Jun 6 [cited 2024 Jun 18];92(12):2171. Available from: /pmc/articles/PMC2361819/
41. Welsh JB, Sapinoso LM, Su AI, Kern SG, Wang-Rodriguez J, Moskaluk CA, et al. Analysis of Gene Expression Identifies Candidate Markers and Pharmacological Targets in Prostate Cancer. [cited 2024 Jun 18]; Available from: http://www.gnf.org/
42. He HC, Bi XC, Zheng ZW, Dai QS, Han ZD, Liang YX, et al. Real-time quantitative RT-PCR assessment of PIM-1 and hK2 mRNA expression in benign prostate hyperplasia and prostate cancer. Med Oncol [Internet]. 2009 Sep 12 [cited 2023 Aug 24];26(3):303–8. Available from: https://link.springer.com/article/10.1007/s12032-008-9120-9
43. Shan M, Xia Q, Yan D, Zhu Y, Zhang X, Zhang G, et al. Molecular analyses of prostate tumors for diagnosis of malignancy on fine-needle aspiration biopsies. Oncotarget. 2017;8(62):104761–71.
44. Yazbek Hanna M, Winterbone M, O'Connell SP, Olivan M, Hurst R, Mills R, et al. Gene-Transcript Expression in Urine Supernatant and Urine Cell-Sediment Are

Different but Equally Useful for Detecting Prostate Cancer. Cancers (Basel) [Internet]. 2023 Feb 1 [cited 2024 Jan 11];15(3). Available from: /pmc/articles/PMC9913640/
45. Mengual L, Ars E, Lozano JJ, Burset M, Izquierdo L, Ingelmo-Torres M, et al. Perfil de expresión génica en el cáncer de próstata: identificación de marcadores candidatos para el diagnóstico no invasivo. Actas Urológicas Españolas. 2014 Apr 1;38(3):143–9.
46. Mahn R, Heukamp LC, Rogenhofer S, Von Ruecker A, Müller SC, Ellinger J. Circulating microRNAs (miRNA) in Serum of Patients With Prostate Cancer. Urology. 2011 May 1;77(5):1265.e9-1265.e16.
47. Figueroa JA, De Raad S, Speights VO, Rinehart JJ. Gene Expression of Insulin-Like Growth Factors and Receptors in Neoplastic Prostate Tissues: Correlation with Clinico-pathological Parameters. http://dx.doi.org/101081/CNV-100000072 [Internet]. 2001 [cited 2023 Aug 24];19(1):28–34. Available from: https://www.tandfonline.com/doi/abs/10.1081/CNV-100000072
48. Heni M, Hennenlotter J, Scharpf M, Lutz SZ, Schwentner C, Todenhöfer T, et al. Insulin Receptor Isoforms A and B as well as Insulin Receptor Substrates-1 and -2 Are Differentially Expressed in Prostate Cancer. PLoS One [Internet]. 2012 Dec 10 [cited 2023 Aug 24];7(12). Available from: /pmc/articles/PMC3519512/
49. Nakamura T, Stephan C, Scorilas A, Yousef GM, Jung K, Diamandis EP. Quantitative analysis of hippostasin/KLK11 gene expression in cancerous and noncancerous prostatic tissues. Urology. 2003 May 1;61(5):1042–6.
50. Yousef GM, Stephan C, Scorilas A, Ellatif MA, Jung K, Kristiansen G, et al. Differential expression of the human kallikrein gene 14 (KLK14) in normal and cancerous prostatic tissues. Prostate [Internet]. 2003 Sep 1 [cited 2024 Jun 18];56(4):287–92. Available from: https://onlinelibrary.wiley.com/doi/full/10.1002/pros.10263
51. Mavridis K, Stravodimos K, Scorilas A. Quantified KLK15 Gene Expression Levels Discriminate Prostate Cancer From Benign Tumors and Constitute a Novel Independent Predictor of Disease Progression. Prostate [Internet]. 2013 Aug 1 [cited 2023 Aug 22];73(11):1191–201. Available from: https://onlinelibrary.wiley.com/doi/full/10.1002/pros.22667
52. Meola J, Goulart LR, Oliveira JDD, Neves AF, Oliveira WP, Saraiva ACM, et al. Differential expression of the KLK2 and KLK3 genes in peripheral blood and tissues of patients with prostate cancer. Genet Mol Biol [Internet]. 2006 [cited 2023 Aug 26];29(2):193–9. Available from: https://www.scielo.br/j/gmb/a/hBtTD6sNTDJpyMfWCPWqqbP/?lang=en
53. Korbakis D, Gregorakis AK, Scorilas A. Quantitative Analysis of Human Kallikrein 5 (KLK5) Expression in Prostate Needle Biopsies: An Independent Cancer Biomarker. Clin Chem [Internet]. 2009 May 1 [cited 2023 Aug 24];55(5):904–13. Available from: https://dx.doi.org/10.1373/clinchem.2008.103788
54. Hellwinkel OJC, Asong LE, Rogmann JP, Sültmann H, Wagner C, Schlomm T, et al. Transcription alterations of members of the ubiquitin–proteasome network in prostate carcinoma. Prostate Cancer Prostatic Dis 2011 141 [Internet]. 2010 Nov 23 [cited 2023 Aug 21];14(1):38–45. Available from: https://www.nature.com/articles/pcan201048
55. Nodouzi V, Nowroozi M, Hashemi M, Javadi G, Mahdian R. Concurrent Down-Regulation of PTEN and NKX3.1 Expression in Iranian Patients with Prostate Cancer. Int Brazilian J Urol Off J Brazilian Soc Urol [Internet]. 2015 [cited 2023 Aug 24];41(5):898. Available from: /pmc/articles/PMC4756965/
56. Tilli TM, Thuler LC, Matos AR, Coutinho-Camillo CM, Soares FA, da Silva EA, et al. Expression analysis of osteopontin mRNA splice variants in prostate cancer and benign prostatic hyperplasia. Exp Mol Pathol. 2012 Feb 1;92(1):13–9.
57. Colombel M, Dante R, Bouvier R, Ribieras S, Pangaud C, Marechal JM, et al.

DIFFERENTIAL RNA EXPRESSION OF THE PS2 GENE IN THE HUMAN BENIGN AND MALIGNANT PROSTATIC TISSUE. J Urol [Internet]. 1999 [cited 2023 Aug 24];162(3 I):927–30. Available from: https://www.auajournals.org/doi/10.1097/00005392-199909010-00092
58. Pampalakis G, Scorilas A, Sotiropoulou G. Novel splice variants of prostate-specific antigen and applications in diagnosis of prostate cancer. Clin Biochem. 2008 May 1;41(7–8):591–7.
59. Talesa VN, Antognelli C, Del Buono C, Stracci F, Serva MR, Cottini E, et al. Diagnostic potential in prostate cancer of a panel of urinary molecular tumor markers. Cancer Biomarkers. 2009 Jan 1;5(6):241–51.
60. Väänänen RM, Lilja H, Cronin A, Kauko L, Rissanen M, Kauko O, et al. Association of transcript levels of 10 established or candidate-biomarker gene targets with cancerous versus non-cancerous prostate tissue from radical prostatectomy specimens. Clin Biochem [Internet]. 2013 May [cited 2023 Aug 24];46(0):670. Available from: /pmc/articles/PMC3628411/
61. Xu LL, Sun C, Petrovics G, Makarem M, Furusato B, Zhang W, et al. Quantitative expression profile of PSGR in prostate cancer. Prostate Cancer Prostatic Dis 2006 91 [Internet]. 2005 Oct 18 [cited 2023 Aug 24];9(1):56–61. Available from: https://www.nature.com/articles/4500836
62. Hu C, Chen B, Zhou Y, Shan Y. High expression of Rab25 contributes to malignant phenotypes and biochemical recurrence in patients with prostate cancer after radical prostatectomy. Cancer Cell Int [Internet]. 2017 Apr 11 [cited 2024 Jun 18];17(1):45. Available from: /pmc/articles/PMC5387234/
63. Engers R, Ziegler S, Mueller M, Walter A, Willers R, Gabbert HE. Prognostic relevance of increased Rac GTPase expression in prostate carcinomas. Endocr Relat Cancer [Internet]. 2007 Jun 1 [cited 2023 Aug 24];14(2):245–56. Available from: https://erc.bioscientifica.com/view/journals/erc/14/2/0140245.xml
64. Louro R, Nakaya HI, Paquola ACM, Martins EAL, Da Silva AM, Verjovski-Almeida S, et al. RASL11A, member of a novel small monomeric GTPase gene family, is down-regulated in prostate tumors. Biochem Biophys Res Commun. 2004 Apr 9;316(3):618–27.
65. Bai VU, Hwang O, Divine GW, Barrack ER, Menon M, Reddy GPV, et al. Averaged Differential Expression for the Discovery of Biomarkers in the Blood of Patients with Prostate Cancer. PLoS One [Internet]. 2012 Apr 6 [cited 2023 Aug 24];7(4). Available from: /pmc/articles/PMC3321043/
66. Zhang DT, Shi JG, Liu Y, Jiang HM. The prognostic value of Smad4 mRNA in patients with prostate cancer. Tumor Biol [Internet]. 2014 Apr 1 [cited 2023 Aug 24];35(4):3333–7. Available from: https://link.springer.com/article/10.1007/s13277-013-1439-y
67. Fritzsche S, Kenzelmann M, Hoffmann MJ, Müller M, Engers R, Gröne HJ, et al. Concomitant down-regulation of SPRY1 and SPRY2 in prostate carcinoma. Endocr Relat Cancer [Internet]. 2006 Sep 1 [cited 2023 Aug 24];13(3):839–49. Available from: https://erc.bioscientifica.com/view/journals/erc/13/3/0130839.xml
68. Ahani M, Mohammad S, Ghaderian H, Azma MM, Kamali K, Gargari BN, et al. Association of STAT3, PTPRT, TNK2-AS1, LINC-ROR Genes Expression Level with Prostate Cancer and Benign Prostatic Hyperplasia. Int J Cancer Manag. 2022;15(1):120188.
69. Nakaya HI, Beckedorff FC, Baldini ML, Fachel AA, Reis EM, Verjovski-Almeida S. Splice variants of TLE family genes and up-regulation of a TLE3 isoform in prostate tumors. Biochem Biophys Res Commun. 2007 Dec 28;364(4):918–23.
70. Salami SS, Schmidt F, Laxman B, Regan MM, Rickman DS, Scherr D, et al. Combining urinary detection of TMPRSS2:ERG and PCA3 with serum PSA to predict diagnosis of prostate cancer. Urol Oncol Semin Orig Investig. 2013 Jul 1;31(5):566–71.
71. Fuessel S, Sickert D, Meye A, Klenk U, Schmidt U, Schmitz M, et al. Multiple

tumor marker analyses (PSA, hK2, PSCA, trp-p8) in primary prostate cancers using quantitative RT-PCR. Int J Oncol [Internet]. 2003 Jul 1 [cited 2024 Jun 18];23(1):221–8. Available from: http://www.spandidos-publications.com/10.3892/ijo.23.1.221/abstract

72. Matos AR, Coutinho-Camillo CM, Thuler LCS, Fonseca FP, Soares FA, Silva EA, et al. Expression analysis of thrombospondin 2 in prostate cancer and benign prostatic hyperplasia. Exp Mol Pathol. 2013 Jun 1;94(3):438–44.

73. Arencibia JM, Martín S, Pérez-Rodríguez FJ, Bonnin A. Gene expression profiling reveals overexpression of TSPAN13 in prostate cancer. Int J Oncol [Internet]. 2009 Feb 1 [cited 2023 Aug 24];34(2):457–63. Available from: http://www.spandidos-publications.com/10.3892/ijo_00000170/abstract

74. Al-Janabi O, Taubert H, Lohse-Fischer A, Fröhner M, Wach S, Stöhr R, et al. Association of tissue mRNA and serum antigen levels of members of the urokinase-type plasminogen activator system with clinical and prognostic parameters in prostate cancer. Biomed Res Int. 2014;2014.

75. Zhang W, Shu P, Wang S, Song J, Liu K, Wang C, et al. ZNF154 is a promising diagnosis biomarker and predicts biochemical recurrence in prostate cancer. Gene. 2018 Oct 30;675:136–43.
